# Atrial Cardiomyopathy in Atrial Fibrillation: Mechanistic Pathways and Emerging Treatment Concepts

**DOI:** 10.3390/jcm14093250

**Published:** 2025-05-07

**Authors:** Paschalis Karakasis, Panagiotis Theofilis, Panayotis K. Vlachakis, Nikolaos Ktenopoulos, Dimitrios Patoulias, Antonios P. Antoniadis, Nikolaos Fragakis

**Affiliations:** 1 Second Department of Cardiology, Hippokration General Hospital, Medical School, Aristotle University of Thessaloniki, 54642 Thessaloniki, Greece; aantoniadis@gmail.com (A.P.A.); fragakis.nikos@googlemail.com (N.F.); 2 First Cardiology Department, School of Medicine, Hippokration General Hospital, National and Kapodistrian University of Athens, 12462 Athens, Greece; panos.theofilis@hotmail.com (P.T.); vlachakispanag@gmail.com (P.K.V.); nikosktenop@gmail.com (N.K.); 3 Second Propedeutic Department of Internal Medicine, Faculty of Medicine, School of Health Sciences, Aristotle University of Thessaloniki, 54124 Thessaloniki, Greece; dipatoulias@gmail.com

**Keywords:** atrial fibrillation, atrial cardiomyopathy, inflammation, fibrosis, metabolic dysfunction, epicardial adipose tissue, rhythm control, substrate modification

## Abstract

Atrial fibrillation (AF) is increasingly recognized not merely as an arrhythmia, but as a clinical manifestation of atrial cardiomyopathy (AtCM)—a progressive, multifaceted disease of the atrial myocardium involving structural, electrical, mechanical, and molecular remodeling. AtCM often precedes AF onset, sustains its perpetuation, and contributes to thromboembolic risk independently of rhythm status. Emerging evidence implicates diverse pathophysiological drivers of AtCM, including inflammation, epicardial adipose tissue, metabolic dysfunction, oxidative stress, ageing, and sex-specific remodeling. The NLRP3 inflammasome has emerged as a central effector in atrial inflammation and remodeling. Gut microbial dysbiosis, lipid dicarbonyl stress, and fibro-fatty infiltration are also increasingly recognized as contributors to arrhythmogenesis. AtCM is further linked to atrial functional valve regurgitation and adverse outcomes in AF. Therapeutically, substrate-directed strategies—ranging from metabolic modulation and immunomodulation to early rhythm control—offer promise for altering the disease trajectory. This review synthesizes mechanistic insights into AtCM and discusses emerging therapeutic paradigms that aim not merely to suppress arrhythmia but to modify the underlying substrate. Recognizing AF as a syndrome of atrial disease reframes management strategies and highlights the urgent need for precision medicine approaches targeting the atrial substrate.

## 1. Introduction

Atrial fibrillation (AF), the most common sustained cardiac arrhythmia, imposes a significant global burden through its associations with stroke, heart failure, cognitive decline, and mortality [1,2,3,4,5,6,7]. Traditionally regarded as a purely electrical disorder, AF is now increasingly recognized as the clinical manifestation of a broader disease entity—atrial cardiomyopathy (AtCM)—characterized by the structural, functional, and molecular remodeling of the atrial myocardium [8,9]. This paradigm shift underscores the concept that AF does not arise de novo but is frequently the culmination of progressive atrial pathological processes.

AtCM encompasses a diverse range of alterations, including fibrosis, myocyte hypertrophy, electrical instability, metabolic dysfunction, and inflammatory activation [10,11]. These abnormalities often precede the onset of AF, persist after restoration of sinus rhythm, and contribute to thromboembolic risk independent of arrhythmia presence. Crucially, AtCM is neither uniform nor monolithic; its phenotypic expression is influenced by a constellation of factors—ageing, metabolic derangements, inflammatory signaling, genetic predisposition, and neurohormonal perturbations—that shape the atrial substrate in a patient-specific manner [12,13,14].

Understanding AtCM as the arrhythmogenic and thrombogenic substrate that underlies AF reframes arrhythmia not merely as an episodic event but as a symptom of a diffuse and evolving myocardial disease. This reconceptualization carries profound therapeutic implications: interventions aimed solely at suppressing AF may be insufficient unless they also address the underlying substrate [15]. Accordingly, the emerging focus has shifted toward a substrate-centric strategy that emphasizes the prevention, reversal, or attenuation of atrial remodeling.

This review synthesizes current mechanistic insights into the development of AtCM and delineates its central role in AF pathophysiology.

## 2. Pathophysiology of AtCM

A growing body of experimental and clinical evidence underscores the intricate pathophysiological relationship between AtCM, AF, and stroke. This triad is interconnected through shared mechanisms—ageing, inflammation, oxidative stress, and atrial stretch—that promote structural, electrical, and autonomic remodeling, culminating in atrial fibrosis and a pro-thrombotic milieu [9]. These maladaptive processes form a self-perpetuating feedback loop, wherein progressive atrial remodeling not only sustains arrhythmogenesis but also amplifies the risk of thromboembolic events. Table 1 summarizes pivotal experimental and clinical studies that have delineated the key molecular, structural, and immunometabolic mechanisms underpinning AtCM in the context of AF.

### 2.1. Inflammasome Activation as a Pathophysiological Driver of AtCM in AF

**Figure 1:**
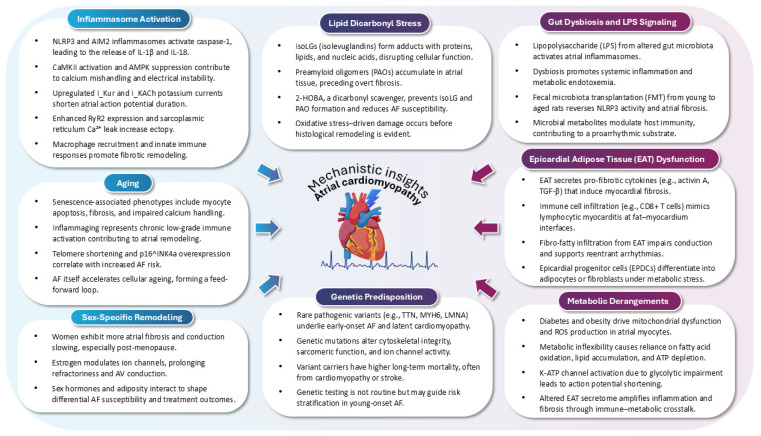
Pathophysiological drivers of atrial cardiomyopathy in atrial fibrillation. Atrial cardiomyopathy (AtCM) arises from a convergence of diverse upstream mechanisms, including inflammasome activation, gut dysbiosis, lipid dicarbonyl stress, epicardial adipose tissue dysfunction, metabolic derangements, ageing, sex-specific remodeling, and genetic predisposition. These processes contribute to the structural, electrical, and thromboembolic remodeling of the atrial myocardium, fostering the initiation and perpetuation of atrial fibrillation (AF).

Emerging evidence has implicated inflammasome signaling, particularly the NLRP3 inflammasome, as a pivotal mediator in the development of AtCM and the promotion of AF (Figure 1) [10]. In murine models, constitutive activation of the NLRP3 inflammasome within atrial tissue has been shown to induce electrophysiological and structural remodeling conducive to AF initiation and maintenance [16,29]. Correspondingly, elevated NLRP3 signaling has been identified in human atrial tissue from individuals with diverse AF phenotypes—including paroxysmal, persistent, and postoperative forms [16,29]—as well as in clinical conditions that predispose individuals to AF, such as obesity, diabetes mellitus, gut microbial dysbiosis, sepsis, and heart failure with preserved ejection fraction (HFpEF) [19,30,31,32,33,34].

The activation process of the NLRP3 inflammasome is characterized by a two-step mechanism involving transcriptional priming and the subsequent oligomeric assembly of its components—NLRP3, the adaptor protein ASC (apoptosis-associated speck-like protein containing a caspase recruitment domain), and pro-caspase-1 [31,32]. Once assembled, the inflammasome facilitates the autocatalytic cleavage of pro-caspase-1 into its active form. Activated caspase-1 subsequently processes pro-inflammatory cytokines interleukin-1β (IL-1β) and interleukin-18 (IL-18) into their biologically active forms and cleaves gasdermin-D, releasing an N-terminal fragment that forms transmembrane pores [31,32]. These pores permit the extracellular release of IL-1β and IL-18, promoting both autocrine and paracrine inflammatory cascades [31,32].

Mice with cardiomyocyte-specific constitutive NLRP3 activation exhibit heightened atrial ectopy and a distinct electrophysiological substrate characterized by action potential duration (APD) shortening—attributed to upregulation of the atrial-selective ultra-rapid delayed rectifier (I_Kur_) and acetylcholine-sensitive inward rectifier (I_K,ACh_) potassium currents—as well as enhanced RyR2 expression and aberrant calcium handling [16]. These electrical alterations co-exist with structural remodeling marked by atrial hypertrophy and interstitial fibrosis [16]. Mechanistically, NLRP3 activation appears to be facilitated by heightened Ca^2+^/calmodulin-dependent protein kinase II (CaMKII) activity and attenuated AMP-activated protein kinase (AMPK) signaling [29,30]. Additionally, lipopolysaccharide derived from gut microbiota has been proposed as a biologically plausible upstream activator of atrial NLRP3 signaling, particularly in the context of ageing [19].

Beyond NLRP3, recent investigations have identified the Absent in Melanoma 2 (AIM2) inflammasome as a novel contributor to AF pathogenesis [17]. In a murine model, high-protein dietary intake was shown to activate AIM2 signaling, leading to a proarrhythmic atrial substrate through mitochondrial oxidative stress and spontaneous calcium release events from the sarcoplasmic reticulum [17]. Collectively, these data underscore the role of inflammasome-mediated innate immune pathways—specifically those involving NLRP3 and AIM2—as central molecular effectors of AtCM and AF, and highlight their potential as therapeutic targets in AF management.

Importantly, recent advances in immunocardiology have highlighted the critical involvement of recruited macrophages in the orchestration of inflammatory signaling pathways that underpin the pathogenesis of AtCM and AF [18]. These findings position macrophage-derived inflammatory cascades not only as mechanistic contributors to atrial remodeling but also as promising targets for therapeutic modulation. Indeed, macrophage-specific interventions are being explored as a novel class of immunomodulatory agents in AF management strategies [35].

Beyond cellular mediators, genetic and epigenetic determinants of innate immune function are increasingly recognized as key modulators of individual susceptibility to AF [36]. By influencing the amplitude and duration of inflammatory responses, these regulatory mechanisms may shape both the initiation and progression of AF [36]. This paradigm underscores the multifactorial nature of inflammation in AF, where inherited and environmentally influenced gene regulation converges on shared immune-inflammatory pathways.

Beyond sterile inflammatory triggers, chronic systemic infections have emerged as underappreciated contributors to atrial cardiomyopathy [37,38]. Persistent low-grade inflammation, immune activation, and endothelial dysfunction associated with chronic infections such as HIV, hepatitis C virus, and cytomegalovirus can promote atrial structural remodeling, fibrotic infiltration, and arrhythmogenic substrate formation [39,40]. In a recent propensity-matched study, HIV-positive patients undergoing AF ablation exhibited a significantly greater burden of left atrial scarring, more frequent non-pulmonary vein triggers, and higher arrhythmia recurrence compared to matched HIV-negative controls, underscoring the arrhythmogenic potential of chronic infection-associated inflammation [41]. Proteomic analyses have further linked HIV infection with subclinical left atrial remodeling and heart failure risk, implicating extracellular matrix turnover, immune checkpoint activation, and cytokine signaling pathways [42]. These findings suggest that chronic systemic infections represent a distinct and clinically relevant inflammatory phenotype of AtCM, warranting further investigation in both diagnostic assessment and therapeutic decision-making.

Concurrently, the concept of “inflammation resolution” has emerged as a critical counter-regulatory process in cardiovascular disease, with implications for halting the transition from acute to chronic inflammation [31]. Specialized pro-resolving mediators (SPMs), such as resolvin D1, have demonstrated efficacy in preclinical models by attenuating the formation of a pro-arrhythmic substrate [43,44]. Specifically, resolvin D1 has shown promise in preventing AF substrate development in animal models of both right-sided pressure overload and left-sided myocardial dysfunction [43,44]. These findings suggest that enhancing endogenous resolution pathways may offer a novel therapeutic axis distinct from conventional anti-inflammatory approaches.

In addition to resolvin D1, other specialized pro-resolving mediators (SPMs), including maresins [45,46] and protectins [47,48,49], have emerged as critical regulators of inflammation resolution. Derived from docosahexaenoic acid (DHA), maresins are produced by macrophages and promote efferocytosis, while limiting neutrophil infiltration [45,46]. Protectins, also DHA-derived, attenuate leukocyte trafficking and cytokine release [47,48,49]. Acting via distinct G-protein coupled receptors, these mediators resolve inflammation without inducing immunosuppression [47,48,49]. Although their role in atrial fibrillation (AF) is not yet fully defined, their capacity to modulate sterile inflammation and fibrosis suggests potential relevance in atrial cardiomyopathy.

Nonetheless, the inflammatory landscape in AF remains heterogeneous. A clear distinction between sterile inflammation—driven by metabolic or mechanical stress—and pathogen-mediated immune responses is lacking. Advancing our understanding of these inflammatory phenotypes will be key to informing targeted immunomodulatory strategies.

### 2.2. Gut Microbiota–Inflammasome Crosstalk in AtCM and AF

The gut microbiota constitutes a complex and metabolically active ecosystem whose composition and functional output are modulated by numerous factors, including diet, physical activity, and other lifestyle influences [50]. Through the production of bioactive metabolites, this microbial community exerts far-reaching effects on host physiology and has been increasingly implicated in the pathogenesis of various cardiovascular disorders, including heart failure, hypertension, obesity, and coronary artery disease [50]. Emerging evidence now extends this paradigm to AtCM and its arrhythmogenic manifestations, highlighting the potential role of gut microbiota-derived signals in the initiation and perpetuation of AF [50].

Among the myriad microbial metabolites, lipopolysaccharide (LPS) and glucose have garnered particular attention as potential mediators of gut dysbiosis-induced atrial remodeling [19]. Mechanistic studies suggest that elevated circulating levels of these metabolites contribute to the structural and electrophysiological remodeling of the atria via activation of the NLRP3 inflammasome signaling pathway. Specifically, dysbiosis-driven NLRP3 activation has been shown to promote the fibrotic remodeling of atrial tissue, thereby facilitating the substrate for AF [19].

In support of a causal relationship, preclinical models have demonstrated that fecal microbiota transplantation (FMT) from young (2–3 months) to aged (22–24 months) rats attenuates age-associated atrial fibrosis and reduces AF susceptibility [31,32]. This effect was mechanistically linked to the suppression of atrial NLRP3 inflammasome activity, providing compelling evidence for the gut–heart axis as a modifiable determinant of arrhythmogenic remodeling [31,32]. Furthermore, selective pharmacologic inhibition of either LPS or the NLRP3 inflammasome recapitulated the protective effects, thereby identifying LPS as a likely upstream effector of inflammasome-mediated atrial remodeling and arrhythmogenesis [31,32].

While these findings underscore the pathogenic potential of gut microbial dysbiosis in creating an inflammatory AtCM phenotype, the precise cellular targets, signaling pathways, and clinical contexts in which this gut–atrial axis is operative remain incompletely defined. Future investigations are warranted to delineate the mechanistic specificity of microbial metabolites in modulating atrial substrates and to identify patient populations in whom microbiota-targeted interventions may hold therapeutic value.

### 2.3. Lipid Dicarbonyl Stress and Preamyloidogenic Injury in AtCM

Inflammatory and oxidative stress states initiate a cascade of lipid peroxidation reactions that generate highly reactive dicarbonyl compounds capable of covalently modifying proteins, nucleic acids, and membrane lipids [51]. These modifications not only disrupt macromolecular function but also foster the formation of cytotoxic amyloidogenic intermediates [51]. Among the most reactive of these lipid-derived electrophiles are isolevuglandins (IsoLGs), a subclass of γ-ketoaldehydes that have emerged as critical effectors of oxidative injury [51,52]. Accumulating preclinical evidence indicates that selective scavenging of IsoLGs mitigates oxidative stress-related damage across various disease contexts, underscoring their pathophysiological relevance and therapeutic tractability [37,53].

In a murine model of hypertension—a condition frequently comorbid with AF—IsoLG-protein adducts and preamyloid oligomers (PAOs) were identified in atrial tissue before the onset of overt structural remodeling or histopathological alterations [54]. This molecular injury profile coincided temporally with heightened AF susceptibility, implicating lipid dicarbonyl stress as an upstream contributor to atrial arrhythmogenesis. Notably, pharmacological administration of the small-molecule dicarbonyl scavenger 2-hydroxybenzylamine (2-HOBA) abrogated the formation of IsoLG-protein adducts, PAO accumulation, and AF vulnerability [54]. In contrast, treatment with the structurally similar but biologically inactive analog 4-hydroxybenzylamine (4-HOBA) failed to confer protection, reinforcing the mechanistic specificity of IsoLG scavenging [54].

These findings advance a novel conceptual framework in which preemptive neutralization of reactive lipid dicarbonyl species, rather than upstream inhibition of reactive oxygen species (ROS) generation, may represent a more precise and effective therapeutic strategy for preventing the molecular sequelae that culminate in AtCM [55]. The clinical translation of this approach is already underway: 2-HOBA has demonstrated a favorable safety and tolerability profile in both preclinical models and Phase I clinical trials, and ongoing studies aim to further evaluate its efficacy in human populations [55].

### 2.4. Epicardial Adipose Tissue as a Modulator of Atrial Substrate and Arrhythmogenesis in AtCM

The accumulation and phenotypic alteration of epicardial adipose tissue (EAT) overlying the atrial myocardium have emerged as critical modulators of AF pathophysiology [56,57,58]. EAT is not merely a passive fat depot but an active paracrine and endocrine organ with the capacity to influence myocardial remodeling through a diverse array of secretory products, including free fatty acids, adipocytokines, peptides, and extracellular vesicles [12,13,59,60,61,62,63]. These bioactive mediators regulate electrophysiologic, fibrotic, and oxidative processes within the adjacent atrial tissue. For instance, factors such as TGFα, KIL-1B, and FABP4 modulate myocardial excitability, while adiponectin exerts antioxidative effects on atrial myocytes [64,65,66].

Pathological conditions such as AF, heart failure, and diabetes alter the EAT secretome, shifting it toward a profibrotic and proinflammatory phenotype. Cytokines, including activin-A and matrix metalloproteinases, are secreted by EAT and facilitate the centripetal spread of fibrosis from the epicardial layer into the adjacent subepicardial myocardium [67,68,69]. Histopathological analyses frequently reveal clusters of mononuclear immune cells—most notably cytotoxic CD8+ T lymphocytes—at the interface between fibrotic regions and EAT, mimicking the lymphocytic myocarditis pattern observed in AtCM [70,71]. Additionally, abnormal EAT secretes a spectrum of chemokines that amplify local inflammation, further contributing to the formation of an arrhythmogenic substrate [72,73].

With disease progression, EAT is progressively replaced by fibrotic tissue, resulting in fibro-fatty infiltration of the subepicardial layers—a phenomenon observed in patients with AF, ageing-related heart failure, or mitral valve disease [20,67,73]. This fibro-fatty remodeling has been firmly established as an arrhythmogenic substrate in both the atria and ventricles, promoting epicardial–endocardial electrical dissociation, localized conduction block, and the emergence of rotors and reentrant circuits [74,75,76]. Computational modeling studies incorporating 3D MR imaging and histo-anatomical data of human atria support the association between fibro-fatty infiltration and conduction slowing, reinforcing its pathogenic relevance [77].

Despite its importance, the regulatory mechanisms governing EAT expansion and phenotypic shifts remain incompletely defined. EAT predominantly arises from epicardial progenitor cells (EPCs), which are quiescent under physiological conditions but can be reactivated in response to local stressors [78,79]. In the human atrium, epicardial-derived cells (EPDCs) reside within the subepicardium and retain the capacity to differentiate into adipocytes or fibroblasts depending on contextual signals. For example, atrial natriuretic peptide (ANP), released by stretched atrial myocytes, promotes EPDC adipogenesis via cGMP-mediated signaling at low concentrations, whereas angiotensin II drives fibrogenic differentiation through the activation of SMAD2/3 and p38-MAPK pathways [80,81]. The adipogenic trajectory of EPDCs is also modulated by lipid availability and the metabolic capacity of EAT to store excess fatty acids, particularly under conditions of dietary lipid surplus [24].

Collectively, these findings delineate EAT as a dynamic contributor to the pathogenesis of AtCM and AF through its involvement in metabolic, inflammatory, and profibrotic signaling. The complex interplay between EPDC differentiation, adipose tissue remodeling, and atrial substrate development highlights EAT as a compelling therapeutic target in the prevention and treatment of AF.

### 2.5. Functional Mitral and Tricuspid Regurgitation and AtCM

Atrial functional mitral and tricuspid regurgitation (AFMR and AFTR, respectively) represent increasingly recognized valvular manifestations of atrial failure and chamber dilatation, distinct from the traditional paradigm of ventricular functional regurgitation [23,82,83,84]. Functional mitral and tricuspid regurgitation (FMR and FTR) broadly refer to valvular incompetence arising not from intrinsic valvular pathology, but from geometric and functional alterations in the cardiac chambers [85]. While ventricular remodeling is the predominant driver of FMR/FTR in most cases, a distinct atrial phenotype—termed AFMR/AFTR—has been delineated, in which the left or right ventricular size and function remain relatively preserved. Instead, annular dilatation secondary to atrial enlargement disrupts leaflet coaptation, giving rise to valvular insufficiency [23,82,83,84].

The clinical and mechanistic understanding of AFMR and AFTR remains incomplete, due in part to heterogeneity in definitions across studies. A recent expert consensus has attempted to standardize diagnostic criteria for AFMR, emphasizing preserved left ventricular (LV) size and systolic function (global and regional) alongside mitral annular dilation and left atrial (LA) enlargement as core features [86]. In more advanced cases, secondary LV dilation may occur, yet the pathophysiologic origin remains rooted in atrial and annular remodeling [87,88]. The principal drivers of this process are AF and HFpEF—two syndromes intimately linked by LA structural and functional remodeling. AF serves as both cause and consequence of progressive atrial dysfunction [88], while chronically elevated LV filling pressures in HFpEF lead to maladaptive atrial remodeling through persistent wall stress. Importantly, AF and HFpEF often coexist, sharing overlapping risk factors and mutually reinforcing pathophysiologic loops.

Rhythm control strategies in patients with AF have demonstrated reductions in LA volume, mitral annular dimension, and severity of AFMR following sinus rhythm restoration, suggesting that atrial unloading and reverse remodeling may be achievable [89,90]. However, these observations remain largely observational, and randomized trials are lacking to confirm whether rhythm control mitigates AFMR or alters clinical outcomes.

Recent studies further underscore the prevalence of occult HFpEF in patients with AF and unexplained dyspnea—particularly in those who remain symptomatic despite successful rhythm control [91,92]. These findings support the emergence of an “AF-predominant” HFpEF phenotype, characterized by disproportionate LA dysfunction and myopathy relative to the degree of LV impairment [93,94]. In this context, AFMR may serve as both a marker and a mediator of hemodynamic compromise. Community-based analyses have shown that only half of patients with moderate to severe isolated AFMR had a documented history of AF [89], indicating that advanced atrial disease may precede or evolve independently of arrhythmia. Notably, even mild to moderate AFMR is associated with adverse outcomes, although this association appears attenuated when adjusting for the severity of LA cardiomyopathy. This finding suggests that the extent of underlying atrial pathology may be more prognostically relevant than AFMR severity alone, while still supporting the potential therapeutic value of targeting AFMR in select HFpEF populations [90].

Importantly, cross-sectional analyses to date have not fully disentangled the temporal and causal relationships among AFMR, LA cardiomyopathy, and HFpEF. The available evidence points toward a bidirectional interplay in which AFMR exacerbates atrial dysfunction while also emerging as a consequence of pre-existing myopathy. Future prospective studies are warranted to clarify these interdependencies and to explore whether intervention on AFMR may offer clinical benefit through the modulation of AtCM and atrial hemodynamics.

### 2.6. Ageing as a Central Driver of AtCM in AF

Ageing represents a fundamental biological process that progressively compromises the structural and functional integrity of the cardiovascular system and is among the most powerful independent risk factors for both AF and stroke [11]. Epidemiological data consistently demonstrate that the prevalence of AF, thromboembolic events, and their shared comorbidities—including diabetes, hypertension, and atherosclerotic vascular disease—increases markedly with advancing age [3]. These risk factors collectively contribute to the development of AtCM, a pathologic substrate characterized by atrial dysfunction, remodeling, and heightened prothrombotic propensity [3].

Mounting evidence supports the notion that AtCM contributes to thrombogenesis not merely through atrial blood stasis but also via endothelial or endocardial dysfunction and systemic hypercoagulability—components of Virchow’s triad [95]. The progressive tissue remodeling intrinsic to AtCM—marked by myocyte apoptosis, compensatory hypertrophy, fibrosis, and autonomic dysregulation—represents a shared endpoint of ageing and other AF-promoting conditions. Intriguingly, AF itself appears to reciprocally accelerate the ageing phenotype of the atrial myocardium, as evidenced by histological and transcriptomic changes consistent with senescence [95].

At the molecular level, several mechanistic pathways link ageing and AtCM, including oxidative stress, dysregulation of intracellular calcium homeostasis, apoptosis, fibrotic remodeling, and alterations in electrophysiological properties [9,96,97]. Despite these insights, a critical knowledge gap remains as to why certain ageing individuals with AtCM progress to clinical AF, while others do not—a question central to personalized risk stratification.

Chronic low-grade inflammation, or “inflammaging”, may represent a unifying mechanism linking ageing with atrial pathology. Inflammaging has been implicated in a spectrum of age-associated disorders such as atherosclerosis, neurodegeneration, sarcopenia, and osteoarthritis [98,99]. Although its origins remain incompletely understood, sustained immune activation may play a pivotal role in facilitating atrial remodeling and arrhythmogenesis in the elderly. Moreover, several biomarkers of cellular senescence—such as telomere attrition, increased expression of p16^INK4a, and SA-β-galactosidase activity—have been associated with AF susceptibility [100]. DNA damage, a principal driver of cellular senescence, has been demonstrated in atrial myocytes subjected to tachypacing and in tissue from patients with persistent AF, further supporting the role of senescence signaling in atrial ageing [21,101].

### 2.7. Sex-Based Differences in AtCM

Epidemiological studies consistently reveal that women exhibit a lower overall prevalence of AF compared to men, a discrepancy that narrows with advancing age [102]. This convergence may reflect sex differences in longevity or age-related factors such as increasing epicardial adiposity in women [102,103,104]. One of the principal contributors to the lower AF prevalence in women appears to be body size—particularly lean body mass—which has been strongly associated with AF risk across both sexes [105]. Despite their lower susceptibility to AF development, women who do develop AF often experience a more severe disease phenotype. Compared with men, they face a disproportionately higher risk of all-cause and cardiovascular mortality [106], as well as more debilitating symptoms and greater reductions in health-related quality of life—suggestive of more extensive structural and functional atrial remodeling [107].

Sex-specific differences are also evident in response to interventional therapies. Multiple studies on catheter ablation have shown that women exhibit higher rates of arrhythmia recurrence following pulmonary vein isolation (PVI) [108], as well as increased risks of periprocedural complications and hospital readmissions [109,110]. A 2022 investigation by Wong et al. [111] sought to elucidate the electrophysiological underpinnings of these disparities. Their findings demonstrated that female patients with AF display more pronounced conduction velocity slowing, a higher prevalence of complex fractionated electrograms, and significantly lower arrhythmia-free survival following both single- and multi-procedure ablation strategies at long-term follow-up—indicators of more advanced atrial remodeling [111].

Hormonal influences may play a pivotal role in mediating these sex-based differences in atrial substrate and arrhythmogenesis. Clinical observations suggest that estrogen deficiency or imbalance—such as during the luteal phase of the menstrual cycle, pregnancy, oral contraceptive use, and menopause—may heighten arrhythmia susceptibility. Estrogen receptors are abundantly expressed in cardiomyocytes, and estrogen administration has been shown to prolong action potential duration, atrioventricular conduction time, and atrial refractoriness. These electrophysiological changes are likely attributable to enhanced calcium influx coupled with the downregulation of repolarizing potassium currents [112,113].

Recent mapping and imaging studies have corroborated the existence of a more fibrotic atrial phenotype in women [114]. Data from the RETAC study, which employed high-density voltage mapping in patients undergoing their first PVI in sinus rhythm, revealed that women—particularly those over 60 years of age with persistent AF—exhibited significantly lower left atrial voltage scores, indicative of extensive low-voltage zones. This sex-based difference was not observed in younger women [114]. Histopathological and cardiac magnetic resonance (CMR) studies have further confirmed that women, regardless of AF status, possess a greater burden of atrial fibrosis compared to their male counterparts [115,116].

Together, these findings suggest that women not only present with a distinct atrial substrate characterized by greater structural remodeling and electrical instability, but also respond differently to both the natural history of AF and its therapeutic interventions. A deeper mechanistic understanding of sex-based atrial remodeling may be essential to optimizing diagnostic, preventive, and therapeutic strategies in AF care.

### 2.8. Metabolic Dysregulation as a Modifiable Determinant of AtCM and AF

Metabolic disorders represent some of the most prevalent and modifiable risk factors implicated in the pathogenesis of AtCM and AF [12,13,60,62,63]. The associations between AF and conditions such as obesity, diabetes mellitus, and metabolic syndrome are robustly supported by epidemiological evidence, whereas the link with liver steatosis-related disorders remains less clearly defined and is the subject of ongoing investigation [117,118]. Data from large population-based cohorts, including the Framingham Heart Study, indicate that diabetes confers a 40% increased risk of AF, with the magnitude of risk correlating positively with disease duration [119,120]. Similarly, elevated body mass index has been consistently associated with increased AF incidence [121].

Mechanistically, metabolic disorders contribute directly to the development and progression of the arrhythmogenic atrial substrate through diverse and interrelated pathways [12,13,60,62,63]. In diabetes and impaired glucose tolerance, atrial cardiomyocytes exhibit metabolic inflexibility characterized by preferential reliance on fatty acid β-oxidation, mitochondrial dysfunction, and excessive generation of reactive oxygen species (ROS) [122]. This maladaptive metabolic profile adversely affects atrial electromechanical properties, contributing to reduced atrial strain and deformation, as demonstrated in clinical studies of diabetic patients [123]. The coupling between cellular metabolism and excitation–contraction processes in atrial cardiomyocytes is tightly regulated, and metabolic perturbations lead to dysfunctional calcium and potassium channel activity [124]. In diabetic models, ATP-sensitive potassium (K-ATP) channels—which are normally closed under physiologic glycolytic conditions—become pathologically activated, likely due to a shift in metabolic substrate preference, thereby promoting action potential shortening and proarrhythmic electrical remodeling [24].

Dietary lipid excess further exacerbates this phenotype by imposing a metabolic overload on atrial myocytes [24]. When fatty acid uptake exceeds mitochondrial oxidative capacity, atrial cells initiate lipid storage pathways characterized by fatty acid elongation, activation of adipogenic transcription programs, and transdifferentiation of progenitor cells into adipocytes [24]. These processes lead to intracellular lipid accumulation and expansion of EAT—an active endocrine organ extensively discussed elsewhere in this article as a contributor to fibrotic remodeling and arrhythmogenesis [125].

Importantly, metabolic disorders alter the secretory phenotype of EAT, particularly in the context of obesity and diabetes, shifting it toward a pro-inflammatory profile enriched in cytokines that facilitate myocardial fibrosis and electrical remodeling [126]. Beyond adipose tissue, immune–metabolic crosstalk involving macrophage activation—well established in ventricular remodeling and atherosclerotic disease—may also serve as a mechanistic bridge between metabolic dysfunction and atrial inflammation [127,128].

These insights underscore the need for interventional studies to assess whether improving metabolic health can modulate the risk or trajectory of AtCM and AF. Targeting metabolic dysregulation may offer a promising avenue for both primary prevention and disease modification in AF management.

### 2.9. Fibrosis as a Key Driver of AtCM in AF

Fibrosis represents a core histopathological hallmark of AtCM, with its extent paralleling AF progression from paroxysmal to long-standing persistent forms [10,129,130,131]. Typically initiating at the pulmonary vein (PV)–left atrial (LA) junction and posterior wall, fibrotic remodeling propagates through the atrial myocardium in a regionally heterogeneous but increasingly confluent pattern [132,133]. This spatial predilection may be explained by factors such as elevated wall stress (per Laplace’s law), mechanical strain from PV inflow, and the distinct embryologic origins of atrial substructures [132,133]. Additionally, MRI data suggest an increased fibrotic burden in posterior LA regions adjacent to the descending aorta, inversely related to aortic proximity [25,134].

Histological studies have revealed that fibrosis often originates in collagen-rich subendocardial and periadventitial zones, progressing into the interstitial space via discrete fibrotic fronts that eventually coalesce [129]. Based on this pattern, fibrosis can be staged from early focal scarring to advanced diffuse fibrosis [135]. Circulating fibrocyte levels have been correlated with fibrotic burden and AF recurrence, supporting their utility as biomarkers [136,137].

Fibrosis involves more than collagen deposition; it includes proliferation and transdifferentiation of mesenchymal cells into α-SMA–positive myofibroblasts and expansion of the ECM with matricellular proteins beyond collagens, such as tenascin-C, biglycan, and thrombospondin-2 [28,138,139,140]. Proteomic analyses have identified decorin—a regulator of collagen fibrillogenesis—as the most altered ECM component in AF, and myeloperoxidase-mediated neutrophil degranulation as a key upstream activator of fibroblast activity and ECM expansion [26,27,141,142]. Transcriptomic studies further implicate epithelial-to-mesenchymal transition and metabolic kinases (e.g., LKB1, AMPK) in regulating profibrotic gene expression and ion channel remodeling [12,62,143]. Notably, early molecular and electrophysiologic changes often precede detectable structural remodeling, suggesting that fibrosis is both a consequence and a driver of AF-related electrical instability [143].

Recent advances in non-invasive and minimally invasive imaging have substantially improved the characterization of atrial fibrosis in atrial cardiomyopathy. Cardiac MRI with late gadolinium enhancement (LGE) enables visualization of localized fibrotic regions, though its precision remains limited by atrial wall thickness and motion artifacts [144,145,146]. Emerging T1 mapping techniques, both native and contrast-enhanced, offer a quantitative assessment of diffuse fibrosis with enhanced reproducibility [25,147]. Complementarily, electroanatomical voltage mapping during ablation provides functional correlates of fibrosis, with low-voltage areas increasingly recognized as electrophysiologic surrogates of structural remodeling [148,149]. The integration of anatomical (LGE/T1) and electrophysiological (voltage) data is advancing risk stratification, individualized procedural planning, and prognostic modeling [146,150]. However, the standardization of imaging protocols and thresholds remains a key barrier to broader clinical implementation.

Together, these findings underscore fibrosis as a dynamic, multifaceted process central to AtCM pathogenesis, with implications for both arrhythmia progression and therapeutic intervention.

### 2.10. Endocardial Remodeling: A Molecular Substrate for Thrombogenesis in AtCM

Beyond fibrotic remodeling, accumulating evidence underscores the importance of endocardial remodeling as a distinct, yet underappreciated, contributor to the pathophysiology of atCM [151,152,153]. Introduced in the 2016 EHRA/HRS/APHRS consensus statement, this concept encompasses structural and molecular alterations of the atrial endothelium that extend the impact of AF beyond electrical dysregulation [154]. Histopathological analyses have identified upregulation of prothrombogenic and inflammatory mediators—such as von Willebrand factor (vWF), vascular cell adhesion molecule-1 (VCAM-1) [155], P-selectin, and monocyte chemoattractant protein-1 (MCP-1)—within the atrial endocardium of patients with AF, independent of comorbid vascular disease [155,156,157]. These changes disrupt the local anticoagulant surface of the endocardium, promote leukocyte adhesion and platelet activation, and facilitate endothelial dysfunction, collectively establishing a thrombogenic microenvironment [158]. Unlike traditional risk models focused on flow stagnation or atrial dilation, endocardial remodeling implicates a localized, molecularly driven substrate of thromboembolic risk. As such, it challenges the sufficiency of conventional anticoagulation paradigms and raises the possibility of precision therapies targeting endothelial inflammation, adhesion molecule signaling, or endothelial-protective pathways [159]. Integrating this dimension into the conceptual framework of AtCM reinforces the notion of AF as a multifaceted atrial disease, with implications far beyond rhythm disturbance alone.

### 2.11. Genetic Underpinnings of Early-Onset AF and AtCM

Emerging data suggest that AF presenting in younger individuals may represent the initial clinical manifestation of genetically driven atrial cardiomyopathy (AtCM), frequently linked to rare pathogenic variants in genes traditionally associated with cardiomyopathy and inherited arrhythmia syndromes [160,161,162,163]. In a large whole-exome sequencing study of 1293 patients with AF onset at ≤65 years (median age 50), pathogenic variants were identified in 10% of individuals, while 63% harbored variants of unknown significance [164]. Notably, the yield was highest in those with AF onset before age 30, with the most commonly implicated genes including TTN, MYH7, MYH6, LMNA, and KCNQ1. During a median follow-up of nearly a decade, carriers of disease-associated variants experienced a 50% increase in all-cause mortality, with deaths attributable to cardiomyopathy, sudden cardiac arrest, and stroke [165].

Complementing these findings, recent developments in polygenic risk scoring (PRS) have introduced new opportunities for early identification of AtCM susceptibility [166,167,168,169]. PRS integrates the cumulative effect of common genetic variants associated with atrial remodeling, fibrosis, conduction abnormalities, and AF risk [170,171]. Large-scale biobank studies have shown that higher PRS correlates not only with AF incidence but also with subclinical atrial structural alterations and increased left atrial volume [171]. When combined with clinical and imaging data, PRS may refine risk stratification, particularly in younger individuals or those with intermediate clinical profiles [172]. Although still in the early stages of clinical adoption, PRS holds promise as a tool for identifying at-risk individuals prior to overt disease onset.

These findings underscore the potential clinical relevance of genetic testing in patients with early-onset AF, particularly in identifying subclinical or evolving forms of cardiomyopathy. Despite growing support for a genetic contribution to AtCM, current clinical guidelines do not endorse routine genetic testing in this context, and insurance coverage remains limited [173]. There is a clear need for prospective investigations and the establishment of a multicenter registry dedicated to atrial-specific cardiomyopathies to advance understanding, improve risk stratification, and guide future diagnostic and therapeutic strategies [174].

## 3. Management Approach

### 3.1. Preventive Strategies Targeting AtCM

**Figure 2:**
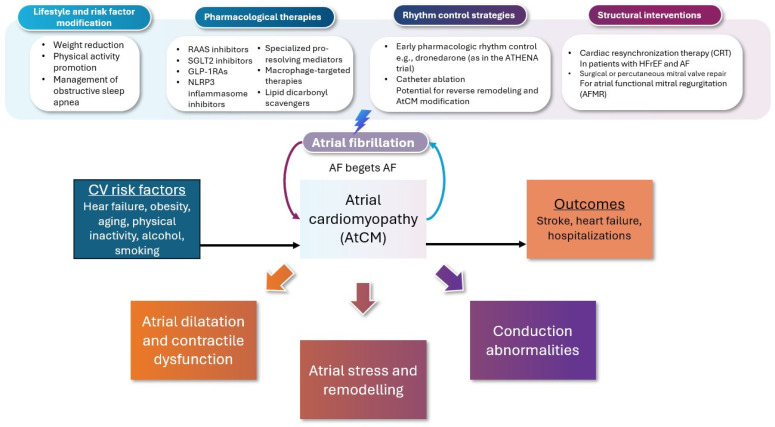
Pathophysiological continuum linking risk factors, atrial cardiomyopathy, atrial fibrillation, and adverse outcomes: therapeutic targets and interventional strategies. Atrial cardiomyopathy arises from the convergence of diverse risk factors—ageing, systemic inflammation, metabolic dysfunction, oxidative stress, and epicardial adipose tissue accumulation—which collectively drive maladaptive atrial remodeling. This remodeling manifests as progressive structural, electrical, and molecular alterations, creating a vulnerable substrate predisposed to the initiation and maintenance of atrial fibrillation. Once established, AF accelerates the underlying myocardial pathology, establishing a self-perpetuating cycle that amplifies thromboembolic risk and contributes to heart failure progression and mortality. Therapeutic strategies span the continuum from upstream prevention to disease modification and downstream rhythm stabilization. Lifestyle interventions, including sustained weight loss and management of sleep-disordered breathing, represent critical upstream targets. Pharmacologic modulation of neurohormonal and metabolic pathways—through RAAS inhibition, SGLT2 inhibitors, and GLP-1 receptor agonists—addresses the core mechanisms of remodeling. Targeted approaches aimed at inflammasome activation, oxidative injury, and fibro-inflammatory signaling offer mechanistically precise avenues for intervention. Rhythm control strategies, particularly early catheter ablation, and structural therapies such as cardiac resynchronization or mitral valve repair, hold promise for reversing remodeling and modifying clinical trajectory. This integrated framework reflects a paradigm shift toward substrate-directed, disease-modifying approaches in the management of atrial fibrillation.

Pathological atrial remodeling, a central feature of AtCM, may be attenuated or potentially reversed by addressing upstream triggers such as pressure or volume overload, or by targeting core molecular mechanisms underlying atrial dysfunction (Figure 2) [175]. The concept of primary prevention—intervening before the overt emergence of AF by preventing or reversing AtCM—is gaining traction, although direct evidence remains limited [175].

Lifestyle modification, particularly sustained weight management, has demonstrated efficacy in reducing AF burden and may prevent the progression of AtCM in patients with established arrhythmia [176,177]. However, evidence supporting lifestyle interventions in the true primary prevention of AtCM—prior to AF onset—is currently scarce. Similarly, while the treatment of obstructive sleep apnea has been associated with improvements in atrial remodeling, its role in the prevention of incident AtCM remains underexplored [178].

Pharmacologic strategies targeting neurohormonal pathways, particularly the renin–angiotensin–aldosterone system (RAAS), have produced mixed results [179,180,181]. Among these, mineralocorticoid receptor antagonists (MRA) have shown the most consistent benefit in reducing AF incidence, suggesting a potential role in modifying early atrial remodeling [182,183]. Nevertheless, the broader utility of RAAS inhibition for the primary prevention of AtCM remains to be conclusively determined.

A major barrier to effective preventive therapy is the current inability to reliably identify the early subclinical stages of AtCM. The development of robust biomarkers and imaging-based tools for early detection will be essential for advancing prevention-focused strategies in AF and AtCM.

### 3.2. Neurohormonal and Metabolic Modulation in Slowing Progression of AtCM in AF

Pharmacologic inhibition of the RAAS has demonstrated modest efficacy in preventing new-onset AF, particularly among patients with underlying heart failure (HF). Meta-analyses of randomized trials have shown that angiotensin-converting enzyme inhibitors and angiotensin receptor blockers reduce the risk of incident AF, though the benefits are less consistent in individuals without structural heart disease [184]. MRA has shown greater efficacy, particularly in reducing recurrent AF and in patients with impaired left ventricular function [184,185]. While these agents modestly reduce LA volume and attenuate fibrotic remodeling in both clinical and experimental models [186], their effects appear less pronounced in populations with paroxysmal AF, likely due to minimal structural remodeling and limited RAAS activation in this setting [178].

Beyond RAAS blockade, sodium–glucose co-transporter 2 (SGLT2) inhibitors—now widely employed in HF management—have emerged as promising agents with pleiotropic cardioprotective effects [187]. Experimental studies demonstrate that SGLT2 inhibition can prevent atrial dilation, fibrosis, mitochondrial dysfunction, and inflammation [188,189,190,191,192,193,194]. Clinically, treatment with SGLT2 inhibitors such as dapagliflozin has been associated with reduced AF incidence in patients with type 2 diabetes, supporting a potential role in atrial substrate modulation.

GLP-1 receptor agonists (GLP-1RAs) exert direct antifibrotic effects by modulating cardiac fibroblast activity through GLP-1 receptor signaling. This suppresses key profibrotic mediators, including TGF-β and connective tissue growth factor, thereby limiting ECM deposition [195,196]. In parallel, GLP-1RAs reduce atrial inflammation via attenuation of macrophage infiltration and cytokine expression, enhance myocardial metabolic efficiency through improved glucose and lipid handling, and bolster antioxidant defenses—all of which contribute to reduced fibrotic remodeling [192,193,197,198,199]. Indirectly, these agents mitigate atrial fibrosis by alleviating cardiac hypertrophy and mechanical wall stress [200,201,202,203].

SGLT2 inhibitors share several antifibrotic properties, including suppression of oxidative stress, inflammation, and neurohormonal activation. Both drug classes also improve endothelial function and reduce angiotensin II and aldosterone levels, blunting pro-fibrotic signaling [188,189,190,191,192,193,204,205,206,207]. While SGLT2 inhibitors primarily relieve atrial strain via diuresis [208], GLP-1RAs attenuate hypertrophic remodeling, offering complementary mechanisms to reduce atrial fibrosis.

Together, these agents may synergistically target multiple fibrogenic pathways, providing a mechanistically comprehensive strategy to mitigate AtCM in high-risk populations. However, further studies are needed to confirm whether these pathophysiological benefits translate into a measurable regression of atrial fibrosis in clinical settings.

### 3.3. Rhythm Control and Reversal of AtCM

While extensive evidence supports the association between atrial remodeling and increased AF burden, as well as the reduced efficacy of rhythm control strategies—including LA ablation—there is comparatively limited documentation linking reversal of remodeling to improved AF-related outcomes. Notably, the ATHENA trial demonstrated that treatment with dronedarone reduced the composite endpoint of first cardiovascular hospitalization or death, and was associated with a lower rate of progression to permanent AF [209,210]. Similarly, the EAST-AFNET 4 trial showed that early rhythm control—primarily pharmacologic—was superior to rate control in reducing adverse cardiovascular events among patients with recently diagnosed AF, particularly those with elevated cardiovascular risk profiles [211,212].

Complementing this, the RAFT-AF trial focused specifically on patients with high-burden AF and concomitant heart failure [213]. Although the primary composite outcome of all-cause mortality and heart failure events narrowly missed statistical significance (HR, 0.71; 95% CI, 0.49–1.03; *p* = 0.066), ablation-based rhythm control led to marked improvements in left ventricular ejection fraction, NT-proBNP levels, exercise capacity, and quality of life metrics compared to rate control [213]. These findings suggest a pathophysiologic link between rhythm control and the reversal or attenuation of atrial cardiomyopathy, particularly when instituted early or in patients with functional compromise.

Collectively, these data advocate a paradigm shift toward proactive rhythm-control strategies as a potential disease-modifying approach in atrial cardiomyopathy.

### 3.4. Interventional Reversal of Atrial Remodeling

#### 3.4.1. Device Therapy

Atrial remodeling, often secondary to elevated intracardiac pressures and volume overload, may be partially reversible following targeted structural interventions. In patients with HFrEF, cardiac resynchronization therapy (CRT) has been shown to reduce LA volume and improve atrial emptying function within months of implantation, primarily as a consequence of left ventricular reverse remodeling and subsequent hemodynamic unloading [214]. Conversely, conduction system pacing—though a more physiological alternative to right ventricular apical pacing—has not demonstrated significant reductions in atrial size in patients with AF and coexisting HFrEF or HFpEF [215,216].

Surgical correction of mitral valve disease also appears to reverse elements of atrial remodeling. In patients with severe mitral regurgitation, mitral valve repair leads to marked reductions in indexed LA volume and improvements in atrial geometry [217]. Similarly, in those undergoing percutaneous mitral commissurotomy, acute reductions in LA pressure are accompanied by corresponding decreases in LA volume and enhancements in atrial conduction properties [218].

#### 3.4.2. Ablation Therapy

Beyond structural interventions, rhythm control strategies—particularly catheter ablation—have demonstrated the potential to modulate atrial remodeling in AF. Structural and electrical remodeling are central to AF pathogenesis and progression, but arrhythmia itself contributes to AtCM, perpetuating a self-reinforcing cycle of disease [219]. Restoration of sinus rhythm remains the most effective strategy to interrupt this feedback loop.

Catheter ablation, as the cornerstone of rhythm control, not only reduces AF burden, but may also reverse atrial remodeling in appropriately selected patients [220,221]. Evidence from voltage mapping and cardiac MRI suggests that rhythm stabilization through ablation is associated with partial reversal of both electrical and structural atrial changes [222]. These findings support the view that ablation may serve as a disease-modifying intervention in patients with AF-induced AtCM.

Taken together, these data highlight the capacity of both structural and rhythm-targeted interventions to mitigate atrial remodeling, particularly when applied early in the course of the disease.

## 4. Conclusions and Future Directions

AtCM represents a paradigm shift in our understanding of AF, reframing arrhythmia as a clinical manifestation of a complex and heterogeneous disease process involving structural, electrical, mechanical, and molecular remodeling of the atrial myocardium. This reconceptualization has profound implications for diagnosis, risk stratification, and treatment, challenging the conventional duration-based classification of AF and supporting a transition toward substrate-guided management.

Therapeutically, early rhythm control—particularly catheter ablation—has demonstrated the potential to reverse elements of AtCM and improve cardiovascular outcomes. Concurrently, pharmacologic agents targeting neurohormonal, metabolic, and inflammatory pathways may offer disease-modifying benefits by attenuating atrial fibrosis and remodeling. Yet, high-quality data from randomized controlled trials remain sparse, and the clinical relevance of reversing AtCM, as opposed to merely controlling rhythm, is still under investigation.

Future research should prioritize the development of a unified, reproducible framework for the diagnosis and staging of AtCM, ideally integrating multimodal data through artificial intelligence and machine learning [223]. Longitudinal studies are needed to assess the temporal evolution of AtCM in relation to AF burden, therapeutic interventions, and thromboembolic risk. Additionally, the identification of AtCM endotypes—defined by specific molecular or imaging signatures—may enable precision medicine approaches and inform targeted treatment strategies.

Ultimately, embedding the concept of AtCM into clinical pathways promises to redefine AF management, shifting the focus from arrhythmia suppression to substrate modification. By advancing early detection, individualized therapy, and mechanistic understanding, this substrate-centric approach may yield durable improvements in AF-related outcomes and prevent the progression of a disease that remains a growing global burden.

## Figures and Tables

**Table 1:** Key studies elucidating the pathophysiological mechanisms of atrial cardiomyopathy in atrial fibrillation.

Author, Year	Model/System	Key Findings	Mechanistic Insight	Implications for AF Pathogenesis
Yao et al., 2018 [16]	Human atrial samples, ATP dog model, CM-specific NLRP3 knock-in, and knockout mice	NLRP3 inflammasome activity is upregulated in atrial cardiomyocytes of patients with AF. Cardiomyocyte-specific activation of NLRP3 promotes atrial ectopy, shortened AERP, Ca^2+^ dysregulation, and structural remodeling. Genetic and pharmacological inhibition of NLRP3 reduces AF susceptibility.	NLRP3 activation in cardiomyocytes leads to increased RyR2-mediated Ca^2+^ leak, enhanced IKur via Kv1.5 upregulation, atrial fibrosis, and reentry substrate formation independent of immune cell infiltration.	Establishes a causative role for cardiomyocyte-intrinsic NLRP3 inflammasome signaling in AF initiation and maintenance. Identifies NLRP3 as a therapeutic target to mitigate electrical and structural remodeling in AF.
Song et al., 2024 [17]	Wildtype and Aim2−/− mice fed high-protein or normal chow diets	High-protein diet (HPD) enhances atrial arrhythmogenesis via AIM2 inflammasome activation. AIM2 deficiency attenuates AF susceptibility, diastolic Ca^2+^ leak, and mitochondrial ROS production.	HPD induces mitochondrial damage, increasing cytoplasmic dsDNA and activating AIM2 inflammasome. This leads to caspase-1 cleavage, IL-1β/IL-18 production, and aberrant Ca^2+^ handling without atrial fibrosis.	Establishes AIM2 as a novel inflammasome pathway promoting AF under dietary stress. Highlights the role of mitochondrial dsDNA and oxidative stress in atrial substrate formation.
Hulsmans et al., 2023 [18]	Human atrial tissue (scRNA-seq), HOMER mouse model, CCR2−/− and Spp1−/− mice	SPP1+ macrophages expand in human AF and HOMER mouse model. Recruited macrophages (via CCR2) and their secretion of SPP1 amplify atrial fibrosis and AF susceptibility. Deleting CCR2 or Spp1 reduces AF inducibility and burden in mice.	Monocyte-derived CCR2+ macrophages express SPP1, promoting fibroblast activation, inflammation, and fibrosis via integrin and CD44 signaling. SPP1 deletion limits macrophage proliferation and fibroblast collagen production.	Demonstrates macrophage-driven immunofibrotic remodeling in AF. SPP1 and CCR2 are highlighted as targets for immunomodulatory therapy in atrial cardiomyopathy.
Zhang et al., 2022 [19]	FMT rat model, primary atrial cells, clinical human data	Aged gut microbiota increases AF susceptibility by promoting LPS and glucose-induced NLRP3 inflammasome activation. Transplantation of aged microbiota induces atrial fibrosis, increased circulating LPS, and impaired glucose tolerance. MCC950 and LPS-RS reduce AF inducibility and fibrosis. Young microbiota transplantation reverses the aged phenotype.	Aged microbiota causes gut barrier dysfunction, elevating circulating LPS. LPS and glucose co-activate NLRP3 inflammasome in CFs via TLR4. This promotes atrial fibrosis through IL-1β/TGF-β1 signaling. CMs are not responsive to this pathway.	Establishes the gut–heart axis in ageing-related AF via microbiota-intestinal barrier–NLRP3 signaling. Highlights microbiota modulation and inflammasome inhibition as therapeutic strategies in elderly AF patients.
Ishii et al., 2021 [20]	Human left atrial appendage tissue and CT imaging in AF patients	Epicardial adipose tissue (EAT) adjacent to atrial myocardium shows greater fibrosis, inflammation, and smaller adipocytes than central EAT. The central-to-marginal adipocyte diameter ratio correlates with EAT and atrial myocardial fibrosis. CT-derived %change in EAT fat attenuation noninvasively reflects fibrotic remodeling.	Proinflammatory cytokines (IL-6, TNF-α, TGF-β1) impair adipogenesis and enhance fibrosis in marginal EAT, which in turn promotes atrial fibrosis. CT imaging detects fibrotic remodeling via increased fat attenuation linked to adipocyte size and fibrosis.	Establishes EAT fibrosis as a profibrotic substrate for AF. Highlights imaging-based biomarkers (fat attenuation change) to detect fibrotic remodeling and stratify AF progression risk.
Adili et al., 2022 [21]	Human LAA tissue, HL-1 atrial cardiomyocytes with tachypacing	Atrial fibrillation induces atrial cardiomyocyte senescence marked by increased SA-β-gal activity, p21/p16/p53 expression, and DNA damage (γH2AX). Senescence burden correlates with early AF recurrence after the maze procedure. Tachypacing induces senescence and SASP in HL-1 cells; siRNA p21 knockdown reduces senescence and restores SR Ca^2+^ protein expression.	Proinflammatory cytokines (IL-6, TNF-α, TGF-β1) impair adipogenesis and enhance fibrosis in marginal EAT, which in turn promotes atrial fibrosis. CT imaging detects fibrotic remodeling via increased fat attenuation linked to adipocyte size and fibrosis.	Identifies atrial cardiomyocyte senescence as a novel contributor to AF progression and recurrence. Suggests that anti-senescence strategies may ameliorate electrical and structural remodeling in AF.
Kuck et al., 2018 [22]	Clinical study (FIRE AND ICE trial, 750 patients with paroxysmal AF)	Female sex was independently associated with a 37% increased risk of AF recurrence and a 36% increased risk of cardiovascular rehospitalization after catheter ablation. Prior DCCV and hypertension were also independent predictors of poor outcomes. History of AF duration predicted repeat ablation.	Sex differences in clinical outcomes may reflect underlying differences in atrial substrate, referral patterns, or hormonal influences. Female patients often had more advanced disease at the time of ablation and were at higher risk for groin complications and procedural adverse events.	Highlights sex-specific vulnerability in AF outcomes, suggesting a potential role for sex-based atrial remodeling. Supports earlier intervention and tailored risk stratification in women with AF.
Guta et al., 2021 [23]	Clinical echocardiographic study (83 AF patients vs. 83 healthy controls)	RA dilation (RAVmin) is the primary determinant of tricuspid annulus (TA) dilation and FTR severity in patients with persistent AF. RV volume and function had limited contribution to FTR. TA area and RAVmin independently predicted FTR grade. TA dilation preceded leaflet tethering.	AF-induced RA remodeling leads to TA dilation, flattening of its saddle shape, and coaptation failure, promoting atriogenic FTR. Right atrial volume (especially RAVmin) is the most reliable indicator of TA geometry and FTR severity.	Supports a direct atriogenic mechanism linking RA dilation and FTR in AF. Emphasizes early rhythm control to prevent progressive RA/TA remodeling and right-sided valve dysfunction.
Perike et al., 2023 [13]	Human RAA tissue, HL-1 atrial cells, lentiviral PPP1R12C mouse model	PPP1R12C expression is upregulated in human AF, promoting PP1c targeting to MLC2a, reducing its phosphorylation, and leading to atrial hypocontractility. Overexpression in mice increases atrial dilation, reduces contractility, and enhances AF susceptibility. MRCK inhibition increases PPP1R12C activity and MLC2a dephosphorylation.	PPP1R12C regulates sarcomeric function by directing PP1c to dephosphorylate MLC2a. This reduces contractility independent of calcium handling. PPP1R12C is modulated by RhoA/MRCK signaling and represents a dynamic node in atrial myofilament remodeling.	Identifies PPP1R12C as a key mediator of atrial hypocontractility and a potential upstream regulator of stroke risk in AF. Therapeutic inhibition may restore contractility and reduce AF recurrence and thromboembolic risk.
Suffee et al., 2022 [24]	C57BL/6J mice subjected to 16-week high-fat diet (HFD) vs. normal diet	HFD mice developed AF vulnerability, atrial dilation, and a distinct atrial metabolic profile with lipid accumulation, enhanced β-oxidation, adipogenesis, and inflammation. Action potentials shortened due to activation of K-ATP channels. HADHA activity and palmitate oxidation increased. Adipogenic markers and immune infiltration (macrophages, T cells) were upregulated in the atria.	HFD induces a shift from glycolysis to fatty acid oxidation in atrial myocardium, activating K-ATP channels and shortening AP duration. FA accumulation triggers adipogenic transformation and low-grade inflammation, contributing to arrhythmogenic substrate.	Links diet-induced metabolic dysregulation to electrical, structural, and immunologic remodeling of atrial myocardium. Supports targeting atrial metabolic pathways as a strategy to prevent obesity-related AF.
Su et al., 2022 [12]	Atrial-specific AMPK α1/α2 knockout mice, in vitro AMPK knockdown in atrial myocytes	AMPK deletion in atrial cardiomyocytes leads to progressive conduction and repolarization abnormalities, atrial ectopy, and spontaneous AF. Electrical remodeling precedes fibrosis and chamber dilation. Pitx2c and Mef2c downregulation mediates transcriptional reprogramming of ion channels and connexins.	AMPK loss alters ion channel and gap junction protein expression (Nav1.5, Kir2.1, Gja1, Gja5) via suppression of Pitx2c and Mef2c. This causes early left atrial conduction delay and prolonged APD, promoting arrhythmogenesis. Right atrial dilation appears early, followed by biatrial fibrosis.	Reveals AMPK as a key homeostatic regulator of atrial electrophysiology and transcriptional identity. Suggests impaired AMPK signaling as a metabolic driver of AF, with potential for upstream preventive therapy.
Hopman et al., 2022 [25]	Clinical study (47 AF patients undergoing pre-ablation LGE-CMR)	Quantification of LA fibrosis using IIR 1.2 and 3SD methods shows significant variation in estimated fibrotic burden (29.80% vs. 8.43%, respectively). Despite good correlation (r = 0.85), agreement is poor (ICC = 0.19), and 34% of patients are reclassified into different fibrosis categories depending on the method. Agreement between CEMRG and ADAS 3D LA was high (ICC = 0.93) when using identical thresholds.	Different fibrosis quantification methods using the LA blood pool as reference produce inconsistent results due to dependence on blood pool signal-to-noise ratio (SNR). This affects both inter-method agreement and patient classification.	Highlights methodological variability in assessing LA fibrosis via LGE-CMR, with implications for risk stratification and treatment selection. Emphasizes the need for standardization and histological validation of fibrosis thresholds.
Kawasaki et al., 2021 [26]	Human left atrial tissue (AF vs. non-AF), proteomics and transcriptomics	Proteomics and GSEA reveal upregulation of neutrophil degranulation, oxidative phosphorylation, and ECM disassembly in AF. NETs and neutrophil granule genes (LCN2, S100A8/9) were elevated in AF tissue. MYH10, required for ciliogenesis, was downregulated in fibroblasts. MYH10 deficiency was associated with impaired primary cilia and fibrotic signaling.	Neutrophil degranulation proteins (MPO, ELANE, MMP9) interlink multiple biological processes (inflammation, oxidative stress, ECM remodeling). Cilium assembly is repressed in AF fibroblasts, potentially impairing antifibrotic signaling via TGF-β and angiotensin II.	Identifies neutrophil degranulation as a hub process coordinating inflammation, metabolism, and fibrosis in AF. Suggests primary cilium loss in fibroblasts contributes to profibrotic remodeling, providing novel mechanistic insight and therapeutic targets.
Meulendijks et al., 2023 [27]	Human EAT and LAA samples (AF, future-onset AF, and non-AF patients), atrial fibroblast culture	EAT secretome from AF patients induces COL1A1 and FN1 gene expression in atrial fibroblasts. Myeloperoxidase (MPO) is the most upregulated protein in EAT and EAT secretome in AF, especially in persistent and future-onset AF. NETs and MPO aggregates localize in fibrofatty infiltrates and subepicardial layers.	EAT-derived neutrophils secrete MPO and NETs, promoting fibroblast activation and ECM remodeling. MPO co-localizes with fibrofatty infiltrates, suggesting involvement in epithelial-to-mesenchymal transition and conduction abnormalities.	Reveals EAT neutrophil activity as a driver of atrial fibrosis and potential early biomarker of AF development. Targets like MPO and neutrophil pathways offer novel therapeutic avenues.
van den Berg et al., 2021 [28]	Clinical cohort (*n* = 150, LAA tissue, blood biomarkers), prospective 2-year follow-up	In patients without prior AF, increased atrial expression of COL1A1, COL3A1, COL8A2, TNC, THBS2, BGN, and EDN1 predicted incident AF. Plasma TNC and COL8A2 correlated with tissue levels and improved AF prediction. Histological remodeling was subtle but included increased epicardial fibrosis and mesenchymal cell content.	Subclinical ECM remodeling with upregulation of collagens, matricellular proteins, and fibroblast-activating factors precedes AF onset. Early transcriptional changes occur before visible interstitial fibrosis, suggesting epigenetic or cellular priming of the atrial substrate.	Establishes that atrial remodeling precedes AF onset in high-risk patients. Identifies early molecular biomarkers (COL8A2, TNC) and gene panels that may guide primary prevention strategies.

Abbreviations: AF, atrial fibrillation; AERP, atrial effective refractory period; APD, action potential duration; ATP, atrial tachypacing; BGN, biglycan; CF, cardiac fibroblast; CM, cardiomyocyte; COL1A1, collagen type I alpha 1 chain; COL3A1, collagen type III alpha 1 chain; COL8A2, collagen type VIII alpha 2 chain; CT, computed tomography; dsDNA, double-stranded DNA; DCCV, direct current cardioversion; ECM, extracellular matrix; EAT, epicardial adipose tissue; EDN1, endothelin-1; FMT, fecal microbiota transplantation; FN1, fibronectin 1; HFD, high-fat diet; HPD, high-protein diet; IIR, image intensity ratio; IL, interleukin; KCNJ2, gene encoding Kir2.1 potassium channel; Kv1.5, voltage-gated potassium channel subunit encoded by KCNA5; LA, left atrium; LAA, left atrial appendage; LAVI, left atrial volume index; LPS, lipopolysaccharide; MLC2a, myosin light chain 2a; MPO, myeloperoxidase; MRCK, myotonic dystrophy kinase-related Cdc42-binding kinase; NETs, neutrophil extracellular traps; NLRP3, NOD-, LRR-, and pyrin domain-containing protein 3; NT-proBNP, N-terminal pro–B-type natriuretic peptide; PP1c, protein phosphatase 1 catalytic subunit; PPP1R12C, protein phosphatase 1 regulatory subunit 12C; RA, right atrium; RAVmin, minimal RA volume; RyR2, ryanodine receptor 2; SASP, senescence-associated secretory phenotype; SCN2B, sodium voltage-gated channel beta subunit 2; SPP1, secreted phosphoprotein 1; TA, tricuspid annulus; TGF-β1, transforming growth factor beta 1; TNC, tenascin-C; THBS2, thrombospondin-2; TLR4, Toll-like receptor 4.

## Data Availability

All data generated in this research are included within the article.
